# Gallbladder sludge and microlithiasis disappearance in a cystic fibrosis patient 1 year after triple combination therapy initiation

**DOI:** 10.1002/ccr3.9481

**Published:** 2024-10-17

**Authors:** Corrado Tagliati, Daniele Veri, Andrea Pietra, Giuseppe Lanni, Davide Battista, Marco Fogante, Giulio Argalia, Cecilia Lanza, Stefano Pantano, Francesca Collini, Maria Di Sabatino, Pietro Ripani

**Affiliations:** ^1^ Radiology, Department of Radiology AST Pesaro Urbino Pesaro Italy; ^2^ Dipartimento dei Servizi UOSD Radiologia Ospedale “San Liberatore” Teramo Italy; ^3^ Genetica Medica, Dipartimento di Scienze Mediche e Chirurgiche IRCCS Azienda Ospedaliero‐Universitaria di Bologna Bologna Italy; ^4^ Radiology, Department of Radiological Sciences Azienda Ospedaliero Universitaria delle Marche Ancona Italy; ^5^ Dipartimento Materno Infantile UOSD CRR Fibrosi Cistica Ospedale “San Liberatore” Teramo Italy

**Keywords:** CFTR modulators, cystic fibrosis, gallbladder, microlithiasis

## Abstract

Gallstones, microlithiasis, gallbladder sludge, and micro‐gallbladder are frequently reported in cystic fibrosis patients, and modulators could modify gallbladder disease, probably reducing biliary secretions viscosity.

## Graphical abstract

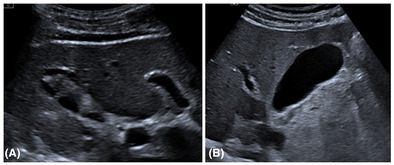

Cystic fibrosis (CF) is a common severe autosomal recessive disorder caused by cystic fibrosis transmembrane conductance regulator (CFTR) gene variants, with the highest prevalence in Europe, North America, and Australia, affecting about 1 in 2000–3000 Caucasian population newborns. The most common disease‐causing gene is the famous F508del, with a frequency of about 70% in CF population.

Over the past three decades, various CFTR treatments that target the underlying defects in CF have been made available to patients. Elexacaftor‐tezacaftor‐ivacaftor (ETI) contains two correctors and a potentiator of the CFTR channel. It is indicated for patients with CF 6 years of age and older who have at least one copy of the F508del gene. Therefore, this treatment provides potential therapy to many patients who had previously been excluded from CFTR modulation therapy.

Progressive lung parenchyma damage caused by recurrent respiratory infections can eventually determine respiratory failure, which is the major cause of death in CF patients. The more prevalent extrapulmonary manifestations are pancreatic insufficiency, chronic rhinosinusitis, liver steatosis, cirrhosis, osteopenia, osteoporosis, and reduced fertility.

Gallbladder abnormalities occur frequently in CF patients, such as gallbladder dysfunction and micro‐gallbladder, and gallstones are found in about 15% of CF patients.^1^ In CF patients, black pigmented stones are more commonly found than cholesterol ones, and cholecystectomy may be performed in up to 4% of patients.^2^ Moreover, bile ducts represent the predominant CFTR liver localization, causing hyperviscous biliary secretions production and cholestasis.^3^ Therefore, CFTR modulators could potentially determine some bile changes. In fact, clinically significant biliary colic, acute cholecystitis, and cholecystectomy shortly after ETI initiation are reported in the literature.

Furthermore, hepatobiliary complications frequency reduction under CFTR modulators was reported.

A 23‐year‐old man with F508del/G85E genotype showed a huge amount of gallbladder sludge and microlithiasis in a small gallbladder under ursodeoxycholic acid treatment. However, 1 year after ETI treatment initiation gallbladder disease disappeared on ultrasound examination, and to the best of our knowledge this is the first case reported in literature (Figure 1). During this first year of treatment, the patient showed mild increase of direct bilirubin and transaminase without biliary symptoms. Moreover, during modulators treatment this patient showed lung and sinus involvement reduction, forced expiratory volume in the first second improvement and forced vital capacity amelioration (Figure 2).

**FIGURE 1:**
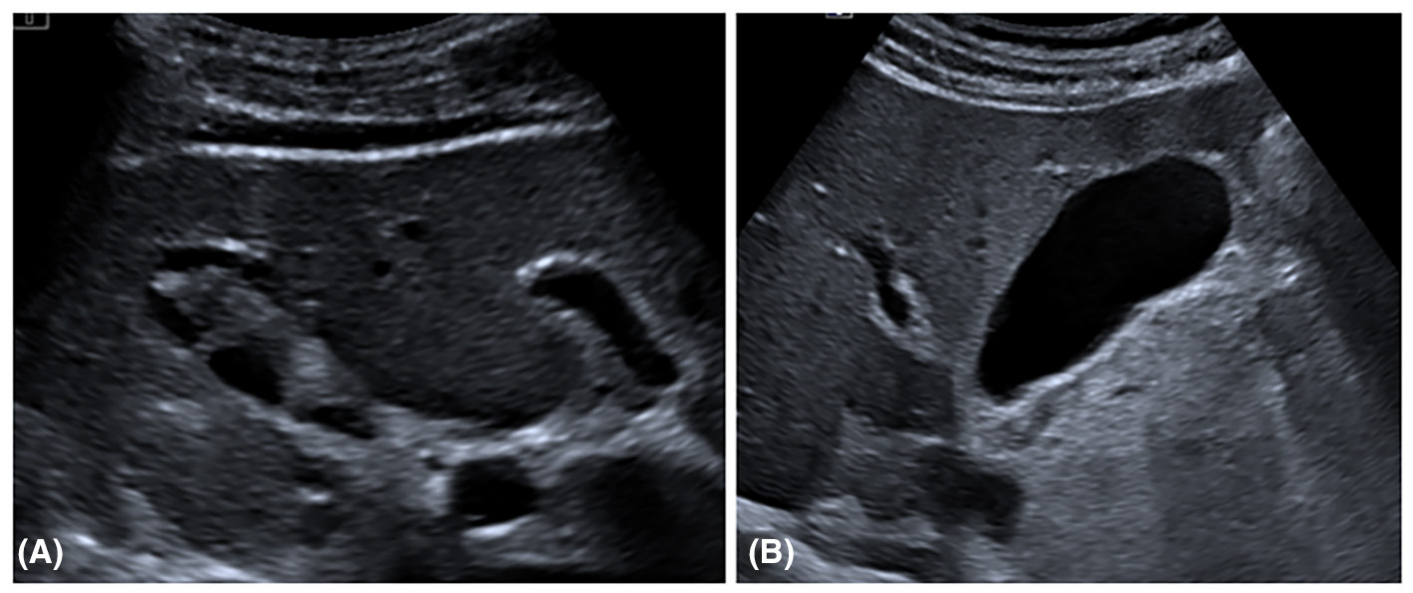
Gallbladder before (A) and 1 year after triple combination therapy initiation where microlithiasis and gallbladder sludge were no more visible (B).

**FIGURE 2:**
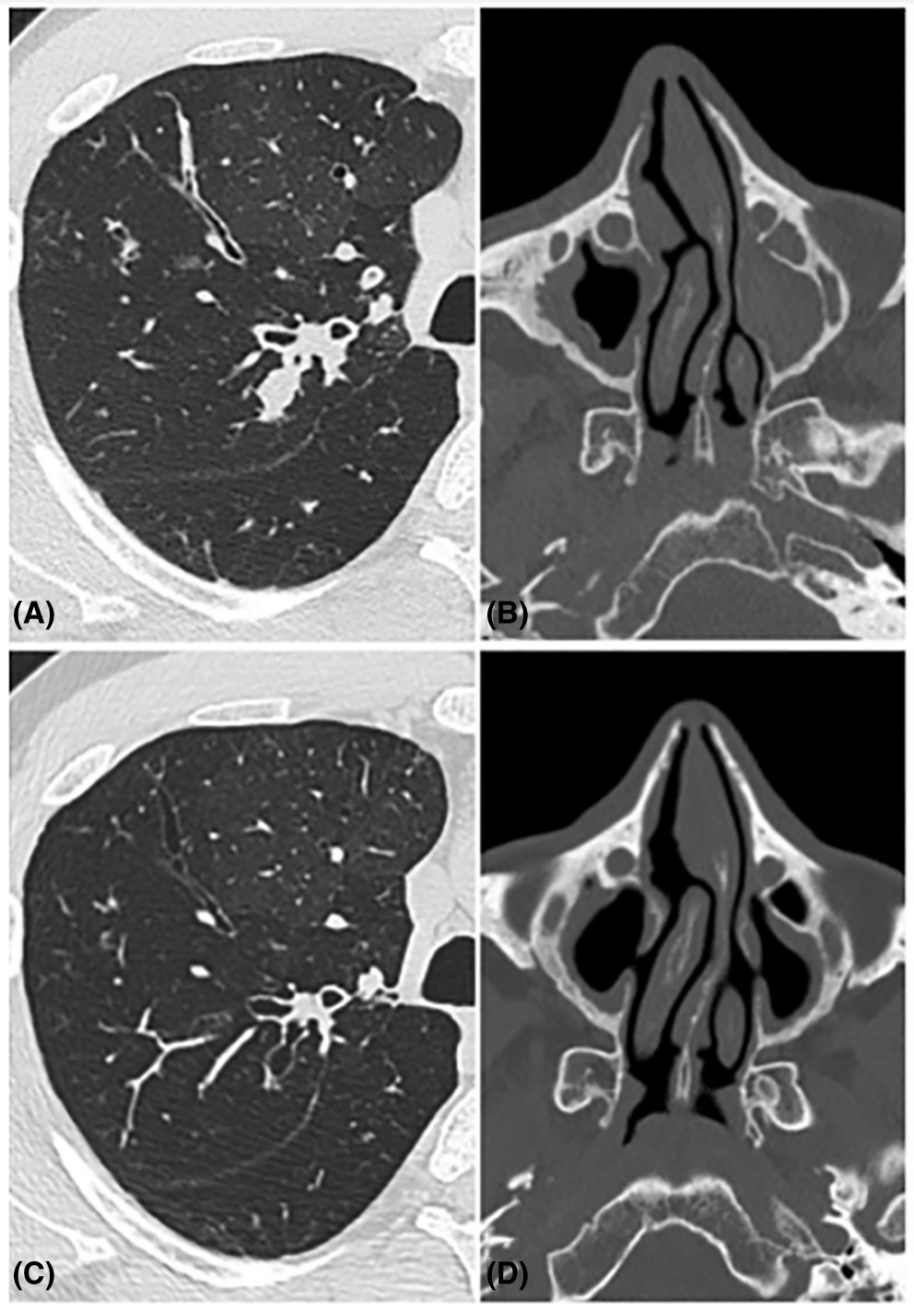
Lung and sinus disease before (A, B) and 1 year after triple combination therapy initiation with evident improvements (C, D).

In conclusion, gallstones, microlithiasis, gallbladder sludge, and micro‐gallbladder are frequently reported in CF patients, and CFTR modulators could modify gallbladder disease, probably reducing biliary secretions viscosity.

## AUTHOR CONTRIBUTIONS

**Corrado Tagliati:** Conceptualization; data curation; methodology; writing – original draft. **Daniele Veri:** Conceptualization; data curation; methodology; writing – original draft. **Andrea Pietra:** Conceptualization; data curation; methodology; writing – original draft. **Giuseppe Lanni:** Conceptualization; data curation; methodology; writing – original draft. **Davide Battista:** Conceptualization; data curation; methodology; writing – original draft. **Marco Fogante:** Conceptualization; data curation; methodology; writing – original draft. **Giulio Argalia:** Supervision; validation; visualization. **Cecilia Lanza:** Supervision; validation; visualization. **Stefano Pantano:** Conceptualization; data curation; methodology; writing – original draft. **Francesca Collini:** Conceptualization; data curation; methodology; writing – original draft. **Maria Di Sabatino:** Conceptualization; data curation; methodology; writing – original draft. **Pietro Ripani:** Supervision; validation; visualization.

## FUNDING INFORMATION

None.

## CONFLICT OF INTEREST STATEMENT

The authors declare no conflicts of interest.

## CONSENT

Written informed consent was obtained from the patient to publish this report in accordance with the journal's patient consent policy.

## Data Availability

The data that support the findings of this study are available from the corresponding author upon reasonable request.
